# Reinstatement of the Chinese endemic species *Styrax zhejiangensis*

**DOI:** 10.3897/phytokeys.133.37942

**Published:** 2019-10-16

**Authors:** Yu-Qing Ruan, Yu-Long Yu, Fen Yu, Guo-Xing Deng, Yu-Lin Liu, Xue-Hui Wu, Ming Tang

**Affiliations:** 1 College of Forestry, Jiangxi Agricultural University, No. 1101, Zhimin Rd., Nanchang, China Jiangxi Agricultural University Nanchang China; 2 Key Laboratory for Bamboo Germplasm Resources and Utilization, No. 1101, Zhimin Rd., Nanchang, China Key Laboratory for Bamboo Germplasm Resources and Utilization Nanchang China; 3 Jiande Forest Farm of Zhejiang, Shiming Rd., Jiande, China Jiande Forest Farm of Zhejiang Jiande China; 4 Management Bureau of Hunan Mangshan National Nature Reserve, Yizhang, China Hunan Mangshan National Nature Reserve Yizhang China

**Keywords:** Flowering phenology, Styracaceae, *Styrax zhejiangensis*, *S. macrocarpus*, synonym

## Abstract

*Styrax zhejiangensis* has been treated as a synonym of *S. macrocarpus*. Examination of herbarium specimens and observation of wild living plants demonstrates that *S. zhejiangensis* is a distinct species and is clearly distinguishable from *S. macrocarpus* through its flowering phenology in which leaves and flowers open simultaneously, its smaller corolla lobes and filaments, and its white-stellate-pubescent seeds. On this basis, we reinstate *S. zhejiangensis* as an accepted species. Photographic images and a distribution map of the two species are provided. A lectotype of *S. zhejiangensis* is also designated.

## Introduction

**Figure 1.**
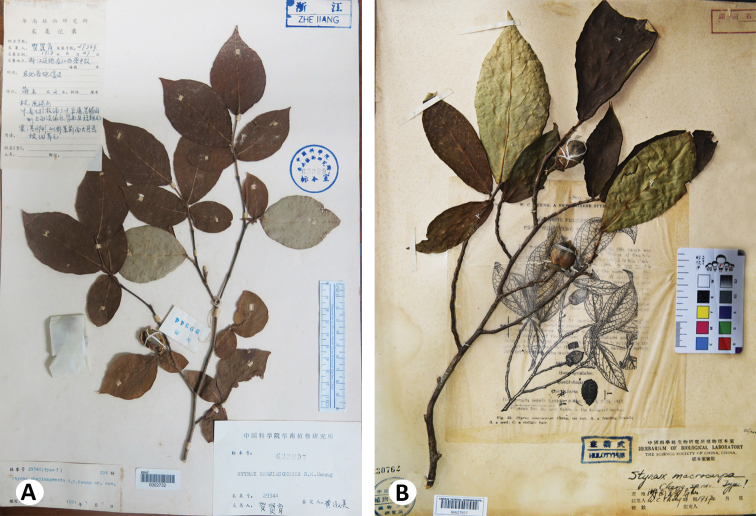
Lectotype sheet of *Styrax zhejiangensis*, China, Zhejiang, Jiande, *X. Y. He 29344* (IBSC) (**A**) and holotype sheet of *S. macrocarpus*, China, Hunan, Yizhang, Mang mountain, *W. C. Cheng s.n.* (PE) (**B**).

**Figure 2.**
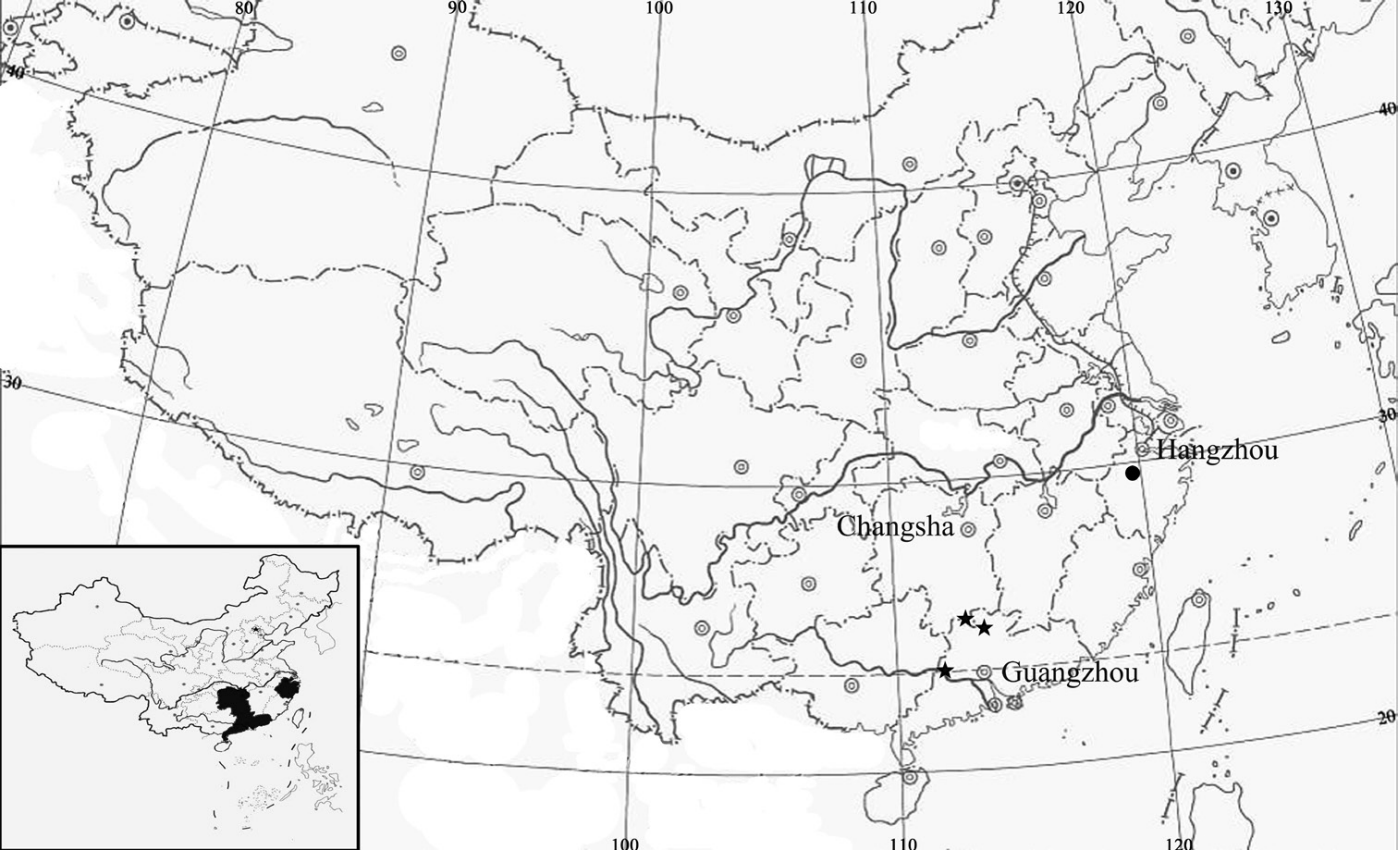
Distribution map of *Styrax zhejiangensis* ([INSERT FIGURE 003]) and *S. macrocarpus*.

*Styrax zhejiangensis* S.M. Hwang & L.L. Yu (Hwang 1983) (Styracaceae) was described on the basis of four specimens of one collection, *X. Y. He 29344* (IBSC, HHBG, NAS; Fig. 1A), from Jiande, Zhejiang Province, China. In the protologue, the authors stated that this species was similar to *S. macrocarpus* Cheng (Cheng 1938) (Fig. 1B), but differed by its bush-like habit, broadly elliptic to ovate-oblong leaves, smaller fruits, and sparsely white-stellate seeds. It was recognized by later authors, including Hwang (1987), Li and Ding (1989), Fang (1992), and Hwang and Grimes (1996), but was synonymized with *S. macrocarpus* by Huang et al. (2003). Huang et al. (2003) purported that there were no essential morphological differences between the two species emphasized by Hwang (1983) other than the presence of stellate trichomes on the seeds in *S. zhejiangensis* versus their absence in *S. macrocarpus*, a difference which they regarded as taxonomically trivial. According to Huang et al. (2003), *S. macrocarpus* sensu lato is a species with a disjunct distribution between southeastern Hunan and western Guangdong (Fig. 2).

After critical examinations of the relevant *Styrax* specimens in major Chinese herbaria, combined with our field observations in the type localities of each entity, we find that *S. zhejiangensis* is a species distinct from *S. macrocarpus*, differing from it in a combination of taxonomically significant morphological characters. Here we provide updated detailed morphological descriptions of these two species, a table of their morphological character differences, a distribution map, photographic images, and conservation assessments.

## Material and methods

Morphological comparisons were made through herbarium studies and field observations. Herbarium studies were conducted in AU, BNU, CSFI, HHBG, IBK, IBSC, JXAU, KUN, NAS and PE. Field observations were made in the type localities of *Styrax zhejiangensis* in Jiande, Zhejiang Province, and *S. macrocarpus* on Mang Mountain, Hunan Province. Moreover, we use IUCN Red List categories (IUCN 2012) to evaluate the conservation status of the two species.

## Results and discussion

**Figure 3.**
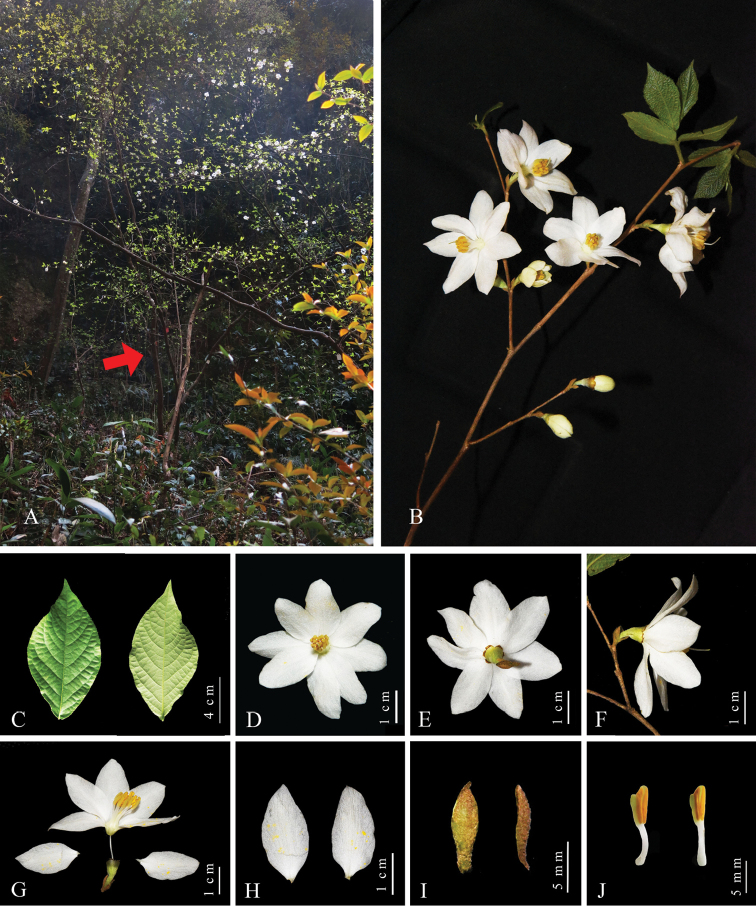
*Styrax zhejiangensis* (Jiande, Zhejiang, China) **A** habitat and habit **B** inflorescence **C** leaf blades **D** flower (top view) **E** flower (back view) **F** flower (side view) **G** opened flower **H** corolla lobes **I** sepals (two sepals pulled apart at the lobe margins of one calyx) **J** stamens. Photographed by Yu-Qing Ruan and Ming Tang.

**Figure 4.**
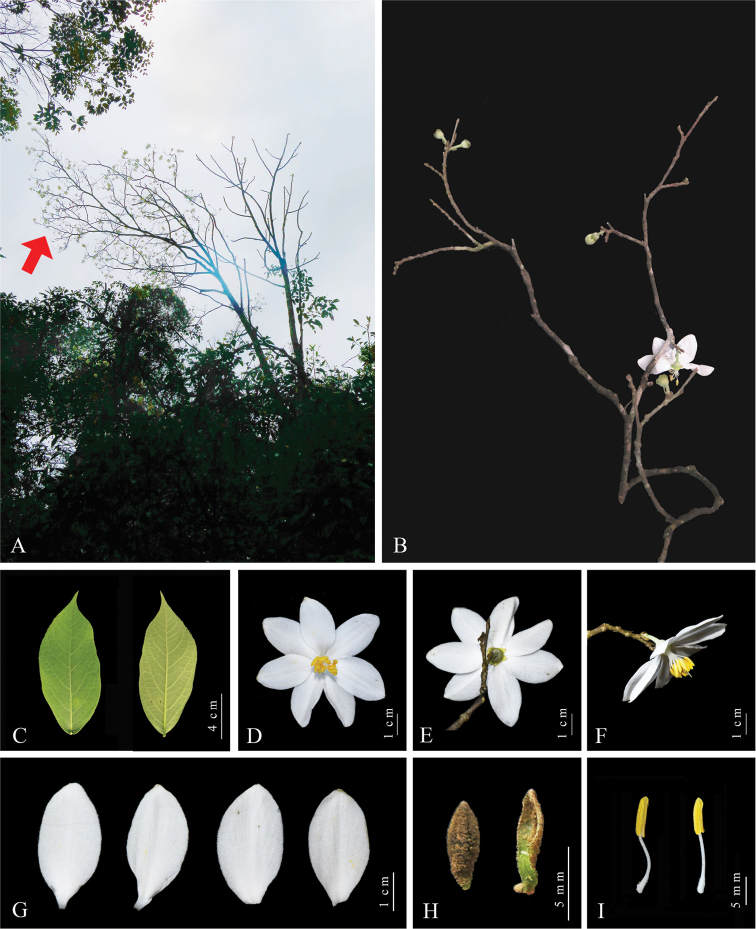
*Styrax macrocarpus* (Yizhang, Hunan, China) **A** habitat and habit; red arrow indicates *S. macrocarpus***B** inflorescence **C** leaf blades in adaxial (left) and abaxial (right) view **D** flower (top view) **E** flower (back view) **F** flower (side view) **G** corolla lobes **H** sepals (two sepals pulled apart at the lobe margins of one calyx) **I** stamens. Photographed by Yu-Qing Ruan and Ming Tang.

**Figure 5.**
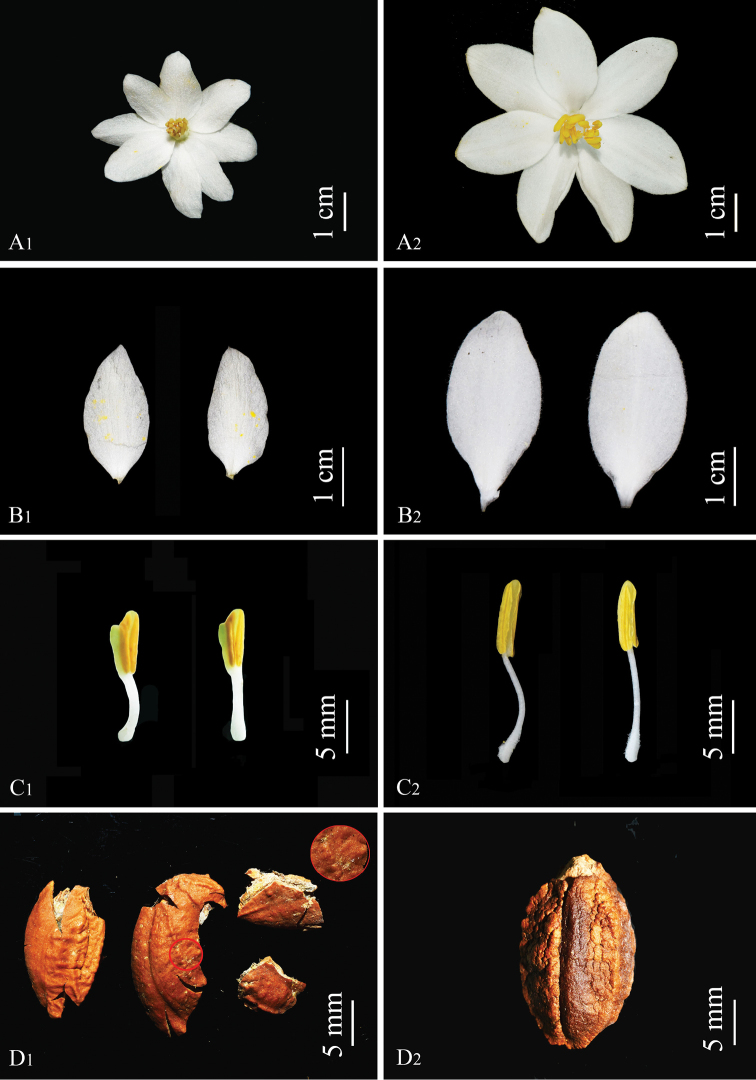
Morphological comparison between *Styrax zhejiangensis* (**A1, B1, C1, D1**) and *S. macrocarpus* (**A2, B2, C2, D2**). **A1, A2** Flower **B1, B2** corolla lobes **C1, C2** stamens **D1, D2** seed (Note the enlarged stellate trichomes in the red circle in figure **D1**). Photographed by Yu-Qing Ruan and Ming Tang.

*Styrax zhejiangensis* differs from *S. macrocarpus* in a combination of morphological characters (Table 1, Figs 3–5). *S. zhejiangensis* produces flowers and leaves simultaneously (vs. flowers before the leaves in *S. macrocarpus*), has smaller flowers (4.0–5.5 cm vs. 6.0–7.5 cm in diameter) with shorter corolla lobes (1.8–2.7 cm vs. 2.8–3.8 cm) and stamens (10–12 cm vs.14–16 cm), and has a seed surface with sparse to dense white-stellate trichomes (vs. glabrous). Furthermore, *S. zhejiangensis* is shrub-like, 1.5–3 m (only one individual reaches 7 m in our observation), with multiple branches from the base, whereas *S. macrocarpus* is tree-like, often over 7 m and with single stem. The pubescence on the corolla also helps to distinguish the two species, with *S. zhejiangensis* having inconspicuous pubescence or being glabrous, and *S. macrocarpus* having conspicuous pubescence.

**Table 1.** Morphological differences between *Styrax zhejiangensis* and *S. macrocarpus*.

**Taxonomic traits**	***Styrax zhejiangensis***	***Styrax macrocarpus***
Stem	1.5–3 (–7) m, shrub-like habit	6–9 (–12) m, tree-like or small tree-like habit
Petiole	Upper 1–3 mm, middle nearly absent	Both upper and middle 2–5 mm
Flowering phenology	Leaves and flowers open simultaneously	Flowers open before leaves
Flower diameter	4.0–5.5 cm	6.0–7.5 cm
Corolla lobe length	1.8–2.7 cm	2.8–3.8 cm
Corolla lobe pubescence	inconspicuous or absent	Conspicuous
Stamens, number	13 to 16	11 to 15
Stamen, length	10–12 mm	14–16 mm
Seeds	with stellate trichomes	without stellate trichomes

Li and Ding (1989) and Hwang and Grimes (1996) described the flowering phenology for *Styrax zhejiangensis* and *S. macrocarpus* and they have been confirmed in our field observation, and undoubtedly serve as a diagnostic character. Li and Ding (1989) also pointed out that the species can also be distinguished by the size of the corolla lobes and numbers of stamens. We observed a substantial difference in the size of their corolla lobes, but no distinct difference in the numbers of their stamens, with 13 to 16 stamens for *S. zhejiangensis* and 11 to 15 for *S. macrocarpus*. Huang et al. (2003) pointed out that the number of stellate trichomes distributed on the seed surface of *S. zhejiangensis* varies from several to dozens, which is consistent with our observation, and they considered that this character by itself cannot be used to distinguish *S. zhejiangensis* from *S. macrocarpus*. However, it should be noted that the seed surface of *S. macrocarpus* is glabrous by our observation, never with stellate trichomes. Thus, combined with the other differences observed, warrant the treatment of *S. zhejiangensis* as an accepted species distinct from *S. macrocarpus*.

### Taxonomic treatment

#### Styrax macrocarpus

Taxon classificationPlantaeEricalesStyracaceae

Cheng (Cheng 1938: 398)

232084B4-FA59-5155-A481-302C11534633

Fig. 1B , 4 , 5 A2, B2, C2, D2

##### Type.

CHINA. Hunan: Yizhang, Mang Mountain, 800 m, 21 August 1937, *W. C. Cheng 7000* [from protologue] (holotype PE00027927!; isotype PE 00027979!)

##### Description.

Trees 6–9(–12) m tall, with a single stem, deciduous. Branchlets subterete, densely gray-brown stellate-pubescent, glabrescent. Leaves alternate, two most proximal leaves on each shoot subopposite to opposite; leaf blade broadly elliptic to ovate-oblong, 5.5–15.0 × 3.0–6.0 cm, papery, glabrous but veins stellate-pubescent, elliptic to obovate-elliptic; apex acute; base cuneate, broadly cuneate or rounded; margin subentire or apically slightly serrate; secondary veins 6 to 10 pairs, tertiary veins subparallel; adaxially plane or slightly sunken, abaxially raised. Petiole 2–5 mm long. Pedicel 9–13 mm long, densely white-stellate-tomentose; bracteoles 6–10 mm long, ovate-lanceolate. Flowers solitary, axillary, opening before leaves. Calyx 5–8 × 6–9 mm, membranaceous, densely gray-stellate-tomentose and sparsely stellate-pubescent; teeth 5 or 6, deltoid, unequal, 2–3 mm, subglabrous. Corolla diameter 6.0–7.5 cm, white, tube 3–4 mm long; lobes 6 or 7, elliptic-obovate, 2.8–3.8 × 1.5–2.5 cm. Stamens 11 to 15, 14–16 mm long, shorter than corolla; filaments 7–10 mm long, basally densely white-stellate-pubescent; anthers 5–7 mm long. Fruit solitary, axillary, ovoid, 2–3 × 2.0–2.5 cm, densely gray-stellate-pubescent, apex shortly pointed. Seeds ovoid-ellipsoid, irregularly rugose, glabrous.

##### Distribution and habitat.

*Styrax macrocarpus* is distributed between southeastern Hunan and western Guangdong (Fig. 2). It grows in sparse forests, valleys or at forest margins at elevations between 130 and 230 m a.s.l.

##### Phenology.

Flowering from mid- to late April and fruiting in August and September.

##### Additional specimens examined.

CHINA. Hunan: Yizhang, *S.H. Chun 2889* (AU, IBK, IBSC, KUN, PE), *5408* (IBSC), *Central South Forestry Institute internship team 02-3 195* (CSFI), *M.S Huang 112743* (IBSC), *H.S. Liao 15727* (CSFI), *P.H. Liang & X.H. Xu 85107* (IBK, IBSC), *S.R. Lin & K.W. Liu 50285* (CSFI), *X.Q. Liu 28884* (IBK, IBSC, NAS, PE), *Y.Q. Ruan & Y.L. Liu 34* (JXAU), *J.G. Xiao 4142* (CSFI), *anonymous 137* (IBSC), *anonymous 1017* (CSFI). Guangdong: Fengkai, *G.L. Shi 14815* (IBSC), *Q.S. Yue 5185* (IBSC); Ruyuan, L. Wu & Y. Tong 3192 (IBSC), Z.L. Chen *30610* (IBSC).

##### Notes.

The two specimens collected from Mengla County, Yunnan (*Y. M. Xia 245*, HITBC) and Yulin, Guangxi (*Y.S. Wu 0289*, IBK) respectively, which were also designated as *Stytax macrocarpus* on the sheets, are confirmed to be wrongly identified and should be *S. chinensis* (Liang 1980: 230), a widely distributed species in the southernmost provinces of China such as Guangdong, Guangxi, Yunnan, Fujian, as well as Southeast Asia. *S. chinesis* could be easily distinguished from *S. macrocarpus* by its bigger (8–23 × 3–12 cm vs 5.5–15.0 × 3.0–6.0 cm), thick-leathery leaves (vs papery) leaves.

#### Styrax zhejiangensis

Taxon classificationPlantaeEricalesStyracaceae

Hwang (Hwang 1983: 75)

C62FB355-698F-58E7-820A-5DB4272B0464

Fig. 1A , 3 , 5A1, B1, C1, D1

##### Type.

CHINA. Zhejiang: Jiande, northeast of Long River, along stream, 27 June 1958, *X. Y. He 29344* (lectotype, here designated, IBSC0002732!; isolectotype IBSC0497542!; isolectotype HHBG-HZ044271!; isolectotype NAS00072216!).

##### Description.

Shrubs, 1.5–3(–7) m tall, often branched at base, deciduous. Branchlets subterete, brown to grayish brown, glabrous. Leaves alternate but subopposite on basal part of branchlet; leaf blade broadly elliptic to ovate-oblong, 2.5–8.0 × 2.0–5.0 cm, papery, adaxially glabrous, abaxially glabrous but veins sparsely stellate-villous, base broadly cuneate to rounded, margin denticulate to subentire, apex acute, secondary veins 5 to 10 pairs, tertiary veins reticulate; adaxially plane or slightly sunken, abaxially raised. Petiole: those of upper leaves 1–3 mm, those of middle nearly sessile. Pedicel 7–13 mm long, densely white-stellate-tomentose; Flowers solitary, axillary, opening simultaneously with leaves. Calyx 5–8 × 5–10 mm, membranaceous; teeth 5 or 6, deltoid, unequal, 1.0–2.5 mm, apex white-glandular-dotted. Corolla diameter 4.0–5.5 cm, white, tube 3–4 mm long; lobes 6 to 8, elliptic-obovate, 1.8–2.7 × 1.0–1.6 cm. Stamens 13 to 16, 10–12 mm long, shorter than corolla; filaments 5–8 mm long, basally densely white-stellate-pubescent; anthers 4–5 mm long. Fruit solitary, axillary, ovoid, 1.8–2.0 × 1.0–1.2 cm, densely gray-stellate-pubescent, apex shortly pointed. Seeds ovoid-ellipsoid, irregularly rugose, sparsely or densely white-stellate-pubescent.

##### Distribution and habitat.

*Styrax zhejiangensis* is only found in Jiande, Zhejiang Province, distributed in Taohuawu, Long River Forest Area (Fig. 2). It grows in sparse forests or at forest margins at elevations between 130 and 230 m a.s.l.

##### Phenology.

Flowering in early April and fruiting in August and September.

##### Additional specimens examined.

CHINA. Zhejiang: Jiande, *G.Y. Li et al. L 0150* (PE); *G.Y. Li et al. L 0154* (PE); *Y.Q. Ruan & Y.L. Liu RL 31* (JXAU); *Y.Q. Ruan & Y.L. Liu RL 46* (JXAU).

### Conservation status of *Styrax zhejiangensis* and *S. macrocarpus*

*Styrax zhejiangensis* is a narrowly distributed species; it is only found in Jiande with a population of less than 100 individuals. Despite the wide distribution of *S. macrocarpus*, it is mainly distributed on Mang Mountain and is not common. According to our observation, the natural regeneration of both of the two species is very poor. Following IUCN Red List categories (IUCN 2012), we categorize *S. zhejiangensis* as critically endangered under criteria B and D and *S. macrocarpus* as endangered under criteria B.

## Supplementary Material

XML Treatment for Styrax macrocarpus

XML Treatment for Styrax zhejiangensis
